# Editorial: Uropathogens, urinary tract infections, the host-pathogen interactions and treatment

**DOI:** 10.3389/fmicb.2023.1183236

**Published:** 2023-03-23

**Authors:** Payam Behzadi, Herney Andrés García-Perdomo, Ana Maria Autrán Gómez, Marina Pinheiro, Meysam Sarshar

**Affiliations:** ^1^Department of Microbiology, Shahr-e-Qods Branch, Islamic Azad University, Tehran, Iran; ^2^Division of Urology, Department of Surgery, School of Medicine, UROGIV Research Group, Universidad del Valle, Cali, Colombia; ^3^Research Office Confederación Americana de Urologia, Madrid, Spain; ^4^Departamento de Ciências Químicas, Faculdade de Farmácia, Universidade do Porto, Porto, Portugal; ^5^Life and Health Sciences Research Institute, Instituto de Investigação em Ciências da Vida e Saúde, Escola de Medicina, Universidade do Minho, Braga, Portugal; ^6^Public Health Unit, ACES Cávado III, ARS Norte, Porto, Portugal; ^7^Research Laboratories, Bambino Gesù Children's Hospital, Istituto di Ricovero e Cura a Carattere Scientifico, Rome, Italy

**Keywords:** uropathogens, bacteria, fungi, urinary tract infections, host-pathogen interactions, treatment

Both Gram-negative and Gram-positive bacteria as well as fungi are responsible for urinary tract infections (UTIs). However, among Gram-negative bacterial agents, uropathogenic *E. coli* (UPEC) is the most common causative agent, followed by uropathogenic *K. pneumoniae* (UPKP) (Behzadi and Behzadi, 2008; Sarshar et al., 2020; Khonsari et al., 2021; Karampatakis et al., 2023). UPEC is armed with a wide range of virulence factors which make it capable to cause different types of UTIs with versatile degrees of severity. UPEC is responsible for 50% of nosocomial and up to 95% of community-acquired UTIs (Behzadi et al., 2016; Behzadi and Behzadi, 2017). Upon the entry of uropathogens (e.g., UPEC pathotypes) into the human urinary system, the invasive bacteria attach to the urothelial cells, internalize and proliferate within the urothelial cells of urinary bladder. Their persistence ability enables them to differentiate into intracellular bacterial communities (IBCs) during early stages of UTIs. Moreover, the recurrent UTIs (rUTIs) are highly rated in patients with UTIs and in particular among females. This feature is associated with those UPEC cells which form quiescent intracellular reservoirs (QIRs) (Behzadi, 2020; Zagaglia et al., 2022). Indeed, early diagnosis of UTIs is crucial for successful treatment because antibiotic-resistant strains have been attributed to the misuse and overuse of antibiotics leading to subsequent treatment failures. In recent years, bacterial resistance mechanisms (e.g., uropathogens) have resulted in serious efforts to find out new therapeutic strategies such as anti-adhesive strategies, microbiota transplantation, nanomaterials, phage therapy, probiotics consumption and vaccination (Sarshar et al., 2020; Zagaglia et al., 2022; Algammal et al., 2023; Mousavifar et al., 2023).

Given the global impact and burden of healthcare-associated infections (e.g., UTIs) on public health, we came to this decision to suggest an impactful Research Topic to be published in the journal Frontiers in Microbiology. Fortunately, we were lucky in our main goal to gather a treasure trove of interesting and updated research studies. Due to this fact, the results of six sharp investigations were published in our Research Topic by 47 international authors. This brilliant collection exposes useful data and information regarding the topic.

In a pilot study, Kafi et al. developed a multiplex high-resolution melting assay (MHRM), a promising tool for simultaneous identification of uropathogens. The results revealed that the MHRM could effectively detect five uropathogenic bacteria including *E. coli, K. pneumoniae, Staphylococcus saprophyticus, Enterococcus faecalis*, and group B streptococci (GBS) directly from the clinical specimens of urine <5 h. Compared to culture, the MHRM shows a specificity range between 99.3 and 100% and a sensitivity of 100% for the all involved bacterial pathogens.

In their study, Huang, Li et al. obtained considerable differences in microbiota composition in female patients with and without rUTI by the advantage of high-throughput next-generation sequencing (NGS) targeting the V3–V4 region of bacterial 16S rRNA. This article provides evidences that some bacterial genera including Corynebacterium, Ralstonia and anaerobes such as Prevotella, Dialister may have significant role in rUTI. Although differential urinary microbiota may play an important role in rUTI, standard urine culture aiming for broadening the range of potentially detectable bacteria imply clinically relevant indicators.

Zhang et al. optimized and validated a metagenomic nanopore sequencing (mNPS) test as a rapid diagnostic method for detection of pathogenic bacteria and antimicrobial resistance genes (ARGs) in clinical samples of urine taken from patients with UTIs. This group compared the consistency of mNPS by recruiting two platforms involving traditional clinical culture and antibiotic sensitivity test (AST). The diagnostic toolkit demonstrated an impressive specificity of 96.8% and a favorable sensitivity of 86.7%, allowing for the rapid and precise detection of UTI pathogens. Although a larger cohort of samples is required, the mNPS diagnostic method has shown promising tool in terms of its speed and accuracy for detecting and predicting uropathogenic pathogens and their antimicrobial resistance patterns.

Another paper published by Huang, Huang et al. systematically compared profiles of causative microbial pathogens and microbial antibiotic resistance patterns isolated from patients with UTIs, in both genders among different age ranges including infants, children, adults and geriatric/aged individuals. In their 12-year observation, *Enterococcus faecium* ranks first as the most important causative microbial agent of UTI among infants, while *E. coli* is the most common etiological microbial agent of UTIs among children, adults and geriatric individuals. In toto, *E. coli, K. pneumoniae, E. faecalis, E. faecium*, and *Pseudomonas aeruginosa* rank first to fifth, respectively, as the five dominated pathogenic bacteria among all age ranges. Importantly, their evidence highlights that uropathogens multidrug-resistance feature rises with age, causing concern among older adults and the geriatric community.

Yang et al. investigated the impact of KguR regulon on UPEC physiology and fitness through transcriptomics and metabolomics approaches. Indeed, the two-component signaling system (TCS) of KguS/KguR is involved in colonization of UPEC in murine urinary system. Yang et al. analyzed the related changes in transcriptomic and metabolomic profiling of kguR mutant compared to the wild type. This study demonstrates that the absence of kguR leads to the differential expression of a significant number of genes related to capsule biosynthesis, iron uptake, acid resistance, amino acid metabolism, and other factors. These variations have a direct impact on the pathobiology of UPEC, therefore employing antagonists targeting TCS could be a viable approach for treating UTIs.

Lastly, Tan et al. assessed how *E. coli* evades Toll-like receptor (TLR) recognition through the regulation of flagellin, which may result in inconsistent of host responses. To explore potential mechanisms underlying the UPEC adaptation and colonization in the urogenital tract, flagellar antigenic variation, their abundance and downstream transcriptional hierarchy were investigated. Their results indicate that neither the recognition of bacterial lipopolysaccharide *via* TLR4-dependent mechanisms nor the growth fitness of the isolates were significant factors in contributing to the variable host responses. The data suggest that selective pressures exist in the urinary tract that allow UPEC strains to maintain motile but exploit population heterogeneity, which may function to prevent host recognition and bacterial killing.

In summary, investigations on uropathogens, their pathogenesis mechanisms, immunogenetics, virulence and predisposing factors in the context of “host-pathogen interactions” can deepen our understanding of the nature of infectious agents. We do hope that the articles published in this topic will be considered as effective guidelines for reducing inaccurate UTIs diagnosis and also provide insights into innovative therapeutic strategies.

## Author contributions

PB, HG-P, AA, MP, and MS have contributed to the writing of the manuscript. All authors have read and agreed to the final version of the manuscript. All authors contributed to the article and approved the submitted version.

## References

[B1] Algammal A. Hetta H. F. Mabrok M. Behzadi P. (2023). Emerging multidrug-resistant bacterial pathogens “superbugs”: a rising public health threat. Front. Microbiol. 4, 246. 10.3389/fmicb.2023.1135614 36819057 PMC9930894

[B2] Behzadi P. (2020). Classical chaperone-usher (CU) adhesive fimbriome: uropathogenic *Escherichia coli* (UPEC) and urinary tract infections (UTIs). Folia Microbiol. 65, 45–65. 10.1007/s12223-019-00719-x 31165977

[B3] Behzadi P. Behzadi E. (2008). The microbial agents of urinary tract infections at central laboratory of Dr. Shariati Hospital, Tehran, Iran. Turk Klin. Tip. Bilim. 28, 445–449.

[B4] Behzadi P. Behzadi E. editors. (2017). Uropathogenic *Escherichia coli*: an ideal resource for DNA microarray probe designing, in Bioinformatics and Biomedical Engineering: 5th International Work-Conference, IWBBIO 2017, Granada, Spain, April 26–28, 2017. Proceedings, Part II 5 (Granada: Springer).

[B5] Behzadi P. Najafi A. Behzadi E. Ranjbar R. (2016). Microarray long oligo probe designing for *Escherichia coli*: an in-silico DNA marker extraction. Central Eur. J. Urol. 69, 105–111. 10.5173/ceju.2016.654 27123336 PMC4846717

[B6] Karampatakis T. Tsergouli K. Behzadi P. (2023). Carbapenem-resistant *Klebsiella pneumoniae*: virulence factors, molecular epidemiology and latest updates in treatment options. Antibiotics 12, 234. 10.3390/antibiotics12020234 36830145 PMC9952820

[B7] Khonsari M. S. Behzadi P. Foroohi F. (2021). The prevalence of type 3 fimbriae in uropathogenic *Escherichia* coli isolated from clinical urine samples. Meta Gene 28, 100881. 10.1016/j.mgene.2021.100881

[B8] Mousavifar L. Sarshar M. Bridot C. Scribano D. Ambrosi C. Palamara A. T.. (2023). Insightful Improvement in the design of potent uropathogenic *E. coli* FimH antagonists. Pharmaceutics 15, 527. 10.3390/pharmaceutics15020527 36839848 PMC9962304

[B9] Sarshar M. Behzadi P. Ambrosi C. Zagaglia C. Palamara A. T. Scribano D.. (2020). FimH and anti-adhesive therapeutics: a disarming strategy against uropathogens. Antibiotics 9, 397. 10.3390/antibiotics9070397 32664222 PMC7400442

[B10] Zagaglia C. Ammendolia M. G. Maurizi L. Nicoletti M. Longhi C. (2022). Urinary tract infections caused by uropathogenic *Escherichia coli* strains—new strategies for an old pathogen. Microorganisms 10, 1425. 10.3390/microorganisms10071425 35889146 PMC9321218

